# Editorial: Ordered and Disordered Cubic Systems: Pyrochlore to Fluorite, Now and the Horizon

**DOI:** 10.3389/fchem.2021.830330

**Published:** 2022-01-12

**Authors:** Gordon James Thorogood, Sarah C. Finkeldei, Maik Kurt Lang, David Simeone

**Affiliations:** ^1^ Australian Nuclear Science and Technology Organization, Sydney, NSW, Australia; ^2^ Nagaoka University of Technology, Nagaoka, Japan; ^3^ Department of Chemistry, University of California, Irvine, Irvine, CA, United States; ^4^ Department of Chemical and Biomolecular Engineering, University of California, Irvine, Irvine, CA, United States; ^5^ Department of Materials Science and Engineering, University of California, Irvine, Irvine, CA, United States; ^6^ Department of Nuclear Engineering, The University of Tennessee, Knoxville, TN, United States; ^7^ CEA,DES,ISAS,DMN, Paris-Saclay, Gif-sur-Yvette, France

**Keywords:** pyrochlore, defect fluorite, disorder-compounds, density-functional theory, fluorite, mineralogy and chemistry

Any internet search for the term pyrochlore, fluorite or the derivation of the two will yield tens of thousands of results. This is clear evidence that the study of these systems, especially since the publication of the landmark review by Subramanian et al. (1983) in 1983 increases with every passing year.

The minerals fluorite and pyrochlore can be thought of as two ends of a continuum that encompass other variations such as defect fluorite. Materials with a fluorite structure are used in a wide range of applications, one reason being its ability to incorporate structural disorder. A cubic structure is the simplest in terms of symmetry and thus can accommodate certain amounts of variation in occupancy of atomic positions over a range of different crystallographic sites. It is this versatility that has seen these humble structures applied to all manner of uses ranging from fuel in a nuclear reactor (Simeone et al., 2017), fuel cell anodes (Lang et al., 2009), ion conductors (Wilde and Catlow, 1998), nuclear waste immobilization (Ewing et al., 2004), thermal barrier coatings (Wilde and Catlow, 1998), catalysts (Kim et al., 2020), and superconductors (Yonezawa et al., 2004) to name only a few. Often there is a common thread in these publications which is investigating in one form or another the order or disorder in those systems with one extreme example being high entropy pyrochlores (Oses et al., 2020).

It was our goal with this special edition to give an overview of what has traditionally been thought to be disorder in pyrochlore, how it relates to defect fluorite, to highlight the newest discoveries as well as the path forward in this research area. We included the effect of order or disorder induced by external means (irradiation, ball milling, pressure, and temperature), induced internally (changes in chemical composition and stoichiometry) and studies that advance the understanding of disorder occurring in those systems and the effect it has on the structure and properties. These studies were planned to encompass experimental, theoretical (such a density functional theory modelling) or a combination of both studies as is common in the current literature.

To place the rest of the articles into context the article by Atencio helps to summarise how definitions have changed over time, what the current nomenclature is and the reasons behind it. In the study the author concludes, and perhaps it is partly a challenge when they state that, surely there are several new pyrochlore- supergroup minerals to be described.

The work of the group from the School of Molecular Sciences and Navrotsky Eyring Center for Materials of the Universe where they examine the order-disorder of several families of cubic oxides from the point of view of how structure, composition, and thermodynamic parameters (enthalpy and entropy) determine the feasibility of different competing ordering processes and structures sets the tone for the special edition (Subramani). This enables the comparison of one structure type directly to another and so provides insight to future work and which of those studies might be most interesting and feasible.

In comparison the paper by Quinn et al. takes a theoretical approach closely aligned with Pauling’s rules for ionic crystal structures and Goldschmidt’s rules for ionic substitution. They propose a simple, but effective, way of determining whether a complex oxide may adopt the disordered fluorite structure and offer guidance in future synthesis.

The study by Kocevski et al. employs a slightly different theoretical approach, modelling from simple point defects to completely disordered structures, the dynamics during the disordering process, and the use of mathematical models to generate ordered solid solution configurations. The results are very similar to those of Quinn et al. and they highlight the challenges of short-range vs long range order and the definition thereof.

One advantage of disorder in pyrochlore and fluorite systems is the flexibility to incorporate elements of varying valence and ionic radius into these solid solutions. To gain further understanding into this phenonium Connor et al. employ DFT+*U* methods to examine the incorporation of U, Np, Pu, Am and Cm actinide elements into pyrochlores, activation energies for oxygen migration and radiation damage-induced structural changes in these materials. Comparison of calculated lattice parameters with experimental results are in very good agreement demonstrating the reliability of the method when applied to these systems.

Doped UO_2_ is interesting being another example of a fluorite structure, that due to its flexibility can accommodate different dopants. Vinograd et al. demonstrate how linking together the thermodynamic and structural models allows the prediction of the lattice parameter as a function of z, T and the oxygen partial pressure, thus providing understanding as to the behaviour of these systems.

In an expansion of the concept of disorder in fluorite systems and how it effects ion conductivity Fossati et al. combine molecular dynamics modelling methods with computational diffraction techniques. Their results demonstrate that defects in the structure and most particularly Frenkel pairs play different roles depending on temperature. Additionally, analysis of the MD simulation boxes indicates that the additional diffraction peaks are the result of local environments with a structure close to the *Pbcn*. In these local environments, the X sublattice has HCP features.

Disorder is typically characterised via either diffraction or spectroscopy techniques. The review by Lumpkin et al. employs transmission electron diffraction to review a range of pyrochlore and defect fluorite type compounds with nominal A_2_B_2_O_7_, A_2_BO_5_, ABC_2_O_7_, and other stoichiometries. Typically, the phase transformations and stability fields in these systems are mapped as a function of the ionic radii of the A and B-site cations, and they highlight situations where this is not the case and outline future investigation.

The review by Wardini et al. on probing disorder in the Transmission Electron Microscope is a must read for any student or early career researcher beginning in the field. It highlights the many methods available to study these systems, for example the section on strain analysis demonstrates how in-depth the mechanisms of disorder in these systems are. As stated in their conclusion “Going forward, as 4D-STEM is increasingly used in tandem with *in situ* and *in operando* studies, the dimensionality of these already rich, multidimensional datasets will extend into the temporal domain, as well as along the axis of the applied stimuli.”

One advantage of pyrochlore being able to accommodate disorder is that cations can adopt valence states seldom reported. Kennedy et al. provide the first report of a stable valence 5 technetium oxide. Displacive disorder of the Pb cations and O(2) anions is observed, as evident from the refinements of diffraction patterns and the Raman spectroscopy. The disorder appears to allow for larger bond lengths for the Technetium thus resulting in it being in the higher valence state.

Finally, there is the work of Finkeldei et al., where the group investigates the formation of defect fluorite via the irradiation of a titanate and zirconate pyrochlore as reported in the literature. This study covers all length scales from neutron diffraction to transmission electron microscopy and somewhat controversially finds that defect fluorite does not form at low irradiation levels, which contradicts much of the literature. They state a second phase is due to the build-up of stress in the sample resulting from the localised amorphization after irradiation via He^2+^ ions. They propose a mechanism for the apparent formation of defect fluorite in X-ray diffraction patterns and it is worth comparing the results of this work with the stress characterisation of Wardini et al., it will be very interesting to see the future response to this work.

In closing, please use this special edition as one point of reference for ordered and disordered cubic systems, with the aim of clarifying the big questions in this area. This work is meant to be used as a cornerstone that may lead to new paths of discovery. Indeed, as we have already shown further development of experimental and modelling techniques have provided an improved understanding in these systems, this will continue in the future and more discoveries can be expected in these systems.
